# The larger attachment glycoprotein of respiratory syncytial virus produced in primary human bronchial epithelial cultures reduces infectivity for cell lines

**DOI:** 10.1371/journal.ppat.1009469

**Published:** 2021-04-08

**Authors:** Tiffany King, Asuncion Mejias, Octavio Ramilo, Mark E. Peeples

**Affiliations:** 1 The Ohio State University College of Medicine, Columbus, Ohio, United States of America; 2 Center for Vaccines and Immunity, The Abagail Wexner Research Institute at Nationwide Children’s Hospital, Columbus, Ohio, United States of America; 3 Department of Pediatrics, The Ohio State University College of Medicine, Columbus, Ohio, United States of America; 4 Division of Pediatric Infectious Diseases, Nationwide Children’s Hospital, The Ohio State University, Columbus, Ohio, United States of America; TWINCORE Zentrum fur Experimentelle und Klinische Infektionsforschung GmbH, GERMANY

## Abstract

Respiratory syncytial virus (RSV) infects the upper and lower respiratory tracts and can cause lower respiratory tract infections in children and elders. RSV has traditionally been isolated, grown, studied and quantified in immortalized cell lines, most frequently HEp-2 cells. However, in vivo RSV infection is modeled more accurately in primary well differentiated human bronchial epithelial (HBE) cultures where RSV targets the ciliated cells and where the putative RSV receptor differs from the receptor on HEp-2 cells. The RSV attachment (G) glycoprotein in virions produced by HEp-2 cells is a highly glycosylated 95 kDa protein with a 32 kDa peptide core. However, virions produced in HBE cultures, RSV (HBE), contain an even larger, 170 kDa, G protein (LgG). Here we show that LgG is found in virions from both subgroups A and B lab-adapted and clinical isolates. Unexpectedly, RSV (HBE) virions were approximately 100-fold more infectious for HBE cultures than for HEp-2 cells. Surprisingly, the cause of this differential infectivity, was reduced infectivity of RSV (HBE) on HEp-2 cells rather than enhanced infectivity on HBE cultures. The lower infectivity of RSV(HBE) for HEp-2 cells is caused by the reduced ability of LgG to interact with heparan sulfate proteoglycans (HSPG), the RSV receptor on HEp-2 cells. The discovery of different infectivity corresponding with the larger form of the RSV attachment protein when produced by HBE cultures highlights the importance of studying a virus produced by its native host cell and the potential impact on quantifying virus infectivity on cell lines where the virus entry mechanisms differ from their natural target cell.

## Author summary

RSV causes severe bronchiolitis in young children, is the leading cause of hospitalization of infants and a major cause of infant mortality in limited-resourced countries. In elders, it rivals influenza virus as a cause of death during the winter months. Many vaccines and antivirals are in development, but none has yet been approved. RSV targets ciliated cells in the upper and lower respiratory tracts. The use of primary HBE cultures previously enabled us to identify the target cell for RSV and a putative receptor on these cells, as well as to better understand the response to RSV infection, all in the cells it infects in vivo. Here, we found that RSV produced in these primary cultures contains a 170 kDa version of its attachment (G) glycoprotein that has lost most of its ability to infect the standard immortalized cell line used to culture RSV, HEp-2 cells, confirming that the receptor used on the two types of cells are different. These findings also demonstrate that significant structural differences with functional consequences can result from the type of cells used to produce the virus.

## Introduction

Approximately 2% of children who are infected with respiratory syncytial virus (RSV) are hospitalized which contributes to a heavy burden of disease across the world [1]. After many decades of research, an effective vaccine or an effective antiviral treatment for RSV remains a hope. For many years, immortalized cells have been used to study RSV because it readily infects and grows in these cells. In the last twenty years, the use of human bronchial epithelial cultures (HBE) have provided an *ex vivo* model for studying RSV pathogenesis.

**Fig 1:**
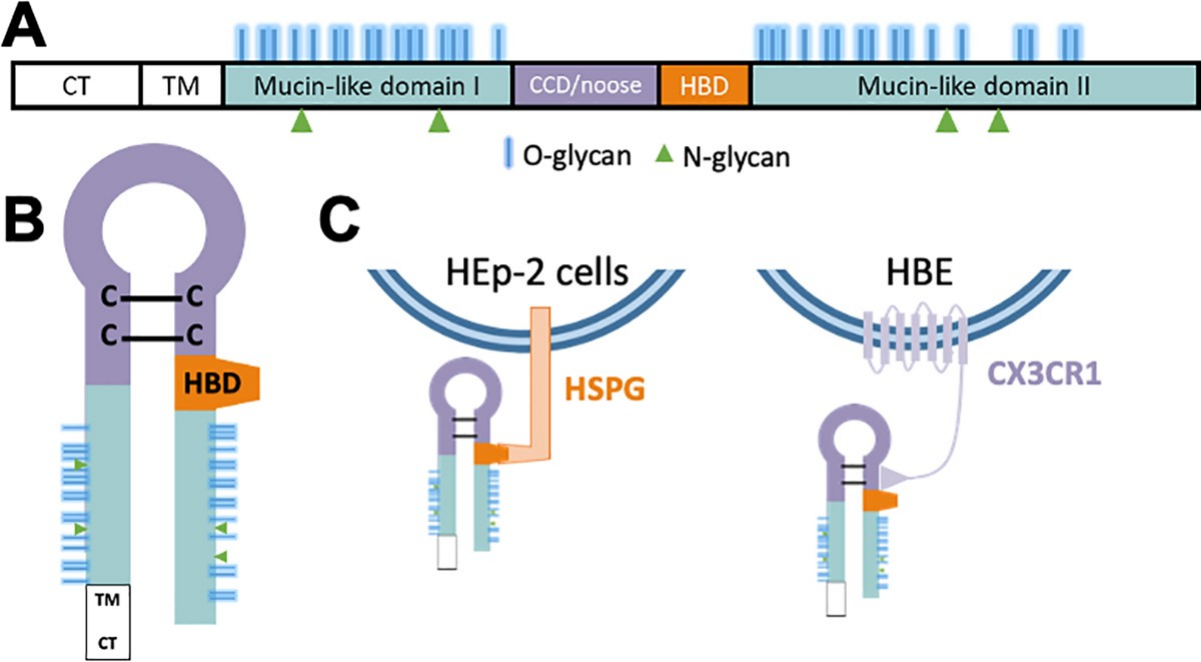
Cartoon of RSV G protein motifs and attachment mechanisms. (A) Domains of RSV G in linear form. (B) Predicted general structure of the RSV G and the likely relative position of its domains. (C) Attachment mechanism of RSV G in immortalized cells and HBE. CT–cytoplasmic tail. TM–transmembrane domain. CCD/noose–central conserved domain/cysteine noose. HBD–heparin binding domain.

The G protein is the attachment glycoprotein of RSV [2]. It is expressed on the surface of virions where it interacts with a target cell receptor to facilitate viral entry. In immortalized cells, the G protein uses heparan sulfate proteoglycans (HSGP) as its receptor. G is important for infection of immortalized cells, however virus without G can still be infectious [3–5]. In contrast, G is necessary for infection in vivo [3–5]. HSPG are not expressed on the apical surface of ciliated epithelial cells where RSV initiates infection of HBE, or in vivo [6]. Instead it appears that CX3CR1, which interacts with the CX3C motif of the G glycoprotein, is a receptor for G in HBE cultures [7–9] (Fig 1C). This chemokine receptor is expressed on the apical surface of ciliated cells in HBE cultures [7–10] and infection can be partially inhibited by monoclonal antibodies to CX3CR1 [7]. Mutations in the CX3C motif of the G protein attenuates RSV infection in HBE cultures and in vivo [7,11,12], modifies the immune response to RSV infection [13] and results in the induction of antibodies that neutralize RSV less efficiently [10].

The G glycoprotein peptide backbone is 32 kDa but the mature G glycoprotein is 90–100 kDa when produced in most cell lines. Post-translational modifications make up nearly two-thirds of its molecular weight [2,14]. G is modified by palmitylation, and N- and O-linked glycosylation producing a fully glycosylated ~95 kDa glycoprotein that is incorporated into budding virions [14,15]. There are 4 predicted N-linked and over 35 O-linked glycosylation sites divided between the two hypervariable mucin-like domains of G (Fig 1B). The processing is conserved in most cell lines with minor size differences likely due to glycosylation [16,17].

Two exceptions to the ~95 kDa fully processed G protein have been described. In Vero cells, G protein is cleaved by cathepsin L resulting in a 55 kDa protein [18]. In HBE, the G protein is produced as a ~170 kDa glycoprotein [19]. Interestingly, in other lung epithelial cell lines such as A549 and primary undifferentiated bronchial epithelial cells, G is 95 kDa not the 170 kDa which appears to be specific for the differentiated cultures [19–21]. Some post-translational modification is likely to be responsible for this increased size of the G protein in HBE. Here we explore the implications of this larger form of the G protein, LgG, and its role in RSV infection of HBE cultures and immortalized cells. We find that LgG has lost its ability to initiate infection of HEp-2 which depends on its interaction with HSPG without altering its ability to infect HBE cultures.

## Methods

### Ethics statement

The collection of clinical samples was approved by the Institutional Review Board (IRB) at Nationwide Children’s Hospital (IRB number 17–00594), classified as a level 1 risk clinical study—no greater than minimal risk (pursuant under 45 Code of Federal Regulations [CFR] 46.404 and 21 CFR 50.51). Informed consent procedures followed in compliance with Nationwide Children’s Research Responsible Conduct Guidelines. Written informed consent obtained from all parents/legal guardians before study participation.

### Primary well differentiated human bronchial epithelial cultures (HBE)

HBE progenitors were isolated from donor airways as described previously, grown for a week to confluency and frozen for later use as described [22]. Progenitor cells were thawed and plated on 0.4 μM pore Transwells (Corning) membranes, 6.5 mm or 12 mm in diameter, fed with ALI medium supplemented with ROCK inhibitor in both the apical and basolateral chambers. Medium in both chambers was replaced with fresh medium every 2–3 days. At 7 days, when the cells were confluent and had formed tight junctions as demonstrated by electrical resistance, the apical medium was removed, and the basal medium was replaced with complete Pneumacult-ALI Medium (STEMCELL Technologies). The medium was replaced with fresh medium and the apical surface was washed with 100 μL of DMEM every 2–3 days for 3 weeks by which time they had become fully differentiated.

### Virus production

Recombinant GFP-expressing rgRSV224 and rgRSV-SF (RSV-ΔG) are both based on the A2 laboratory strain of RSV (19). RSV-B1 was purchased from ATCC (Manassas, VA). Clinical RSV isolate strains A2001/2-20 (2–20) and A2001/3-12 (3–12) were obtained through BEI Resources from NIAID. Clinical isolate strains NCH-232 (232) and NCH-894 (894) were collected from patients at Nationwide Children’s Hospital and passaged in HEp-2 cells. Passage 4 was used for these experiments. RSV stocks were grown in HEp-2 cells [RSV(HEp-2)] in DMEM-10% fbs (fetal bovine serum). GFP expressing viruses were serially diluted and titrated on HEp-2 cells by inoculating for 2 h at 37°C and counting GFP foci at 24 hpi. Viruses and cells were determined to be mycoplasma-free using the ATCC Mycoplasma detection kit. Other RSV strains were titrated similarly but quantified after fixing infected cells in 4% paraformaldehyde, permeabilizing with 0.1% Triton X-100 in PBS for 20 min, staining with L9 (anti-G) monoclonal antibody followed by goat anti-mouse IgG-Texas Red (Vector Labs, Burlingame, CA) and counting foci. Detection of infected cells by GFP expression or by fluorescent immunostaining are equally sensitive.

To produce RSV stocks on HBE, [RSV(HBE)], well differentiated HBE cultures growing on 12 mm Transwells were inoculated at moi 0.01 for 4 h at 37°C. After incubating at 37°C, 5% CO_2_ for 48 h, RSV(HBE) was collected from the apical surface by washing with 250 μL of DMEM supplemented with 10% fbs for 1–2 h at 37°C. RSV(HBE) containing medium was pooled, vortex briefly and centrifuged at 14,000 g for 10 min to remove debris. Supernatant was centrifuged at 20,000 g for 90 min at 4°C, virus pellets were resuspended in fresh DMEM 10% fbs, aliquoted and snap frozen on dry ice for storage at -80°C. RSV(HEp-2) was prepared similarly for infectivity comparison to RSV(HBE).

### HBE/HEp-2 calculation

All virus stocks were titrated on HEp-2 cells. To compare RSV infectivity on HEp-2 cells with infectivity on HBE, both RSV(HEp-2) and RSV(HBE) were diluted to 100 ffu in 50 μL for inoculation of both HEp-2 and HBE, and RSV(HBE) were diluted 1:500 for inoculation of HBE cultures. These dilutions were determined based on preliminary experiments. HEp-2 cells on 96-well plates and well differentiated HBE cultures on 6.5 mm transwell filters with 0.4 μm pores (Corning, Glendale, AZ) were inoculated with 50 μL of viruses of the appropriate dilutions for 4 h at 37°C. All infections were performed in triplicate. After incubation fresh media was replaced on HEp-2 cells and viral inoculum removed from the HBE cultures. GFP expressing cells were counted at 20 hpi for HBE and 24 hpi for HEp-2 cells. HBE/HEp-2 ratios were calculated based on the same day experiment.

### Western blotting

Viral stocks were pelleted by centrifugation at 20,000 g for 90 minutes at 4°C. The virus pellets were re-suspended in lysis buffer and prepared for gel electrophoresis with NuPage LDS sample buffer (Thermo) and 10% BME. Samples were applied to a 4–12% NuPage Tris-Bis pre-cast gel (Thermo) using MOPS running buffer (Thermo) electrophoresed at 170 V for 75 to 100 min. Proteins were transferred to a nitrocellulose membrane (Thermo) using the iBlot dry system. Membranes were blocked using LiCor Odyssey PBS blocking buffer, incubated with L9 monoclonal antibody for 1 h, washed with PBS 0.02% Tween20, then incubated with secondary anti-Mouse-800CW antibody (LiCor) for 1 h. Membranes were analyzed and imaged using with the LiCoR imaging system.

### RT-qPCR

RNA from viral inoculum was extracted on the same day as HEp-2 and HBE culture infections using QIAmp Viral Mini Kit (Qiagen). High Capacity cDNA kit (Thermo) and qPCR was used to reverse transcribe 10 μL of extracted RNA. Quantitative PCR was performed using the Primtetime Assay (IDT) with primers and probes designed to target the nucleocapsid gene. Forward primer: 5’-GGGAGAGGTAGCTCCAGAATA-3’, reverse primer sequence: 5’-CTCCTAA TCACGGCTGTAAGAC-3’ and Probe sequence: 5’-TCCACAATCAGGAGAGTCATGCC-3’. qPCR was performed using Applied Biosciences OneStepPlus. Virus copy number was determined using a standard curve of purified linearized cDNA plasmid containing the RSV genome. We found no significant difference in genome calculations when using random or genome specific primers for reverse transcription.

### Glycosaminoglycan (GAG) dependency index

CHO-K1 and CHO-A745 obtained from ATCC were plated in 96-well plates. CHO-A745 are a CHO-K1 derived cell line deficient in xylosyl transferase [23]. Virus titrations were performed on both sets of cells on the same day. GFP infected cells were counted 24 hpi. Viral titers were calculated for each virus on each cell type. The GAG dependency index was calculated by dividing the titer on CHO-K1 by the titer on CHO-A745, as previously described [19].

### Clinical samples

Three clinical samples, IN-04, IN-156 and OUT-07, were obtained from infants hospitalized or evaluated in the outpatient setting with RSV infection. The IN-04 specimen was collected by nasopharyngeal swab and IN-156 and OUT-07 by nasal wash. All samples were sequenced to determine the genotype of ON-1. Specimens were aliquoted and stored at -80°C and thawed at 37°C for experiments. Each virus was grown on well-differentiated HBE (12 mm) cultures as described above.

## Results

### Virions produced in HBE cultures by both RSV subtypes contain a 170 kDa LgG glycoprotein

**Fig 2:**
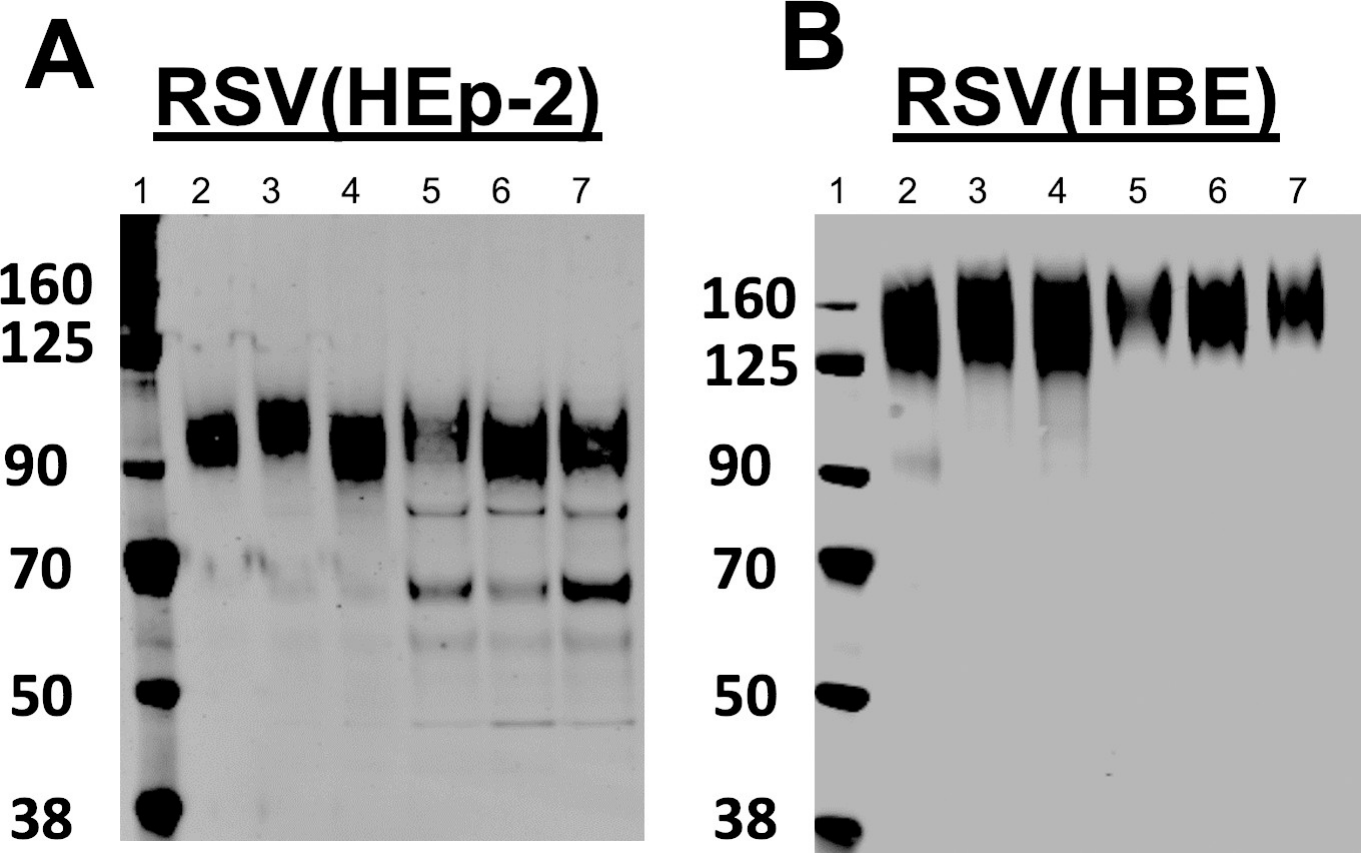
RSV G in laboratory and clinical isolate viruses grown in HEp-2 and HBE. At 5 dpi, RSV was collected from HEp-2 medium (A) or an apical wash of HBE (B), pelleted by centrifugation and analyzed by gel electrophoresis with SDS and 10% BME and immunoblotted with mAb L9 against G. Subgroup A: A2, 2–20, 3–12 (Lanes 2–4). Subgroup B: B1, 2.32, 894 (Lanes 5–7).

We previously demonstrated that RSV infected HBE produce virus with a G glycoprotein that is much larger than the same protein produced in HEp-2 cells, approximately 170 kDa vs. 95 kDa [19]. To determine if this larger G (LgG) protein is unique to the A2 laboratory strain, we determined the size of the G protein from laboratory and clinical isolates from both RSV subgroups A and B, produced in HBE. We chose well-characterized subgroup A clinical isolates, 2–20 and 3–12, and recently collected subgroup B clinical isolates: NCH-232 and NCH-894. We inoculated HEp-2 and HBE cultures with each of these viruses, incubated cells until CPE was visible, collected the medium, clarified by low speed centrifugation and pelleted the virions by high speed centrifugation. Proteins were separated by SDS-PAGE, blotted, and probed with the monoclonal antibody (mAb) L9. L9 recognizes the central conserved domain (CCD) of the G protein and residues in the neck of the cysteine noose [7]. The G protein from all four virion types migrated at 170 kDa, regardless of subgroup or specific virus isolate (Fig 2), or the individual donor for the HBE cells (not shown).

### RSV grown in HBE cultures is more infectious for HBE compared to HEp-2 cells

**Fig 3:**
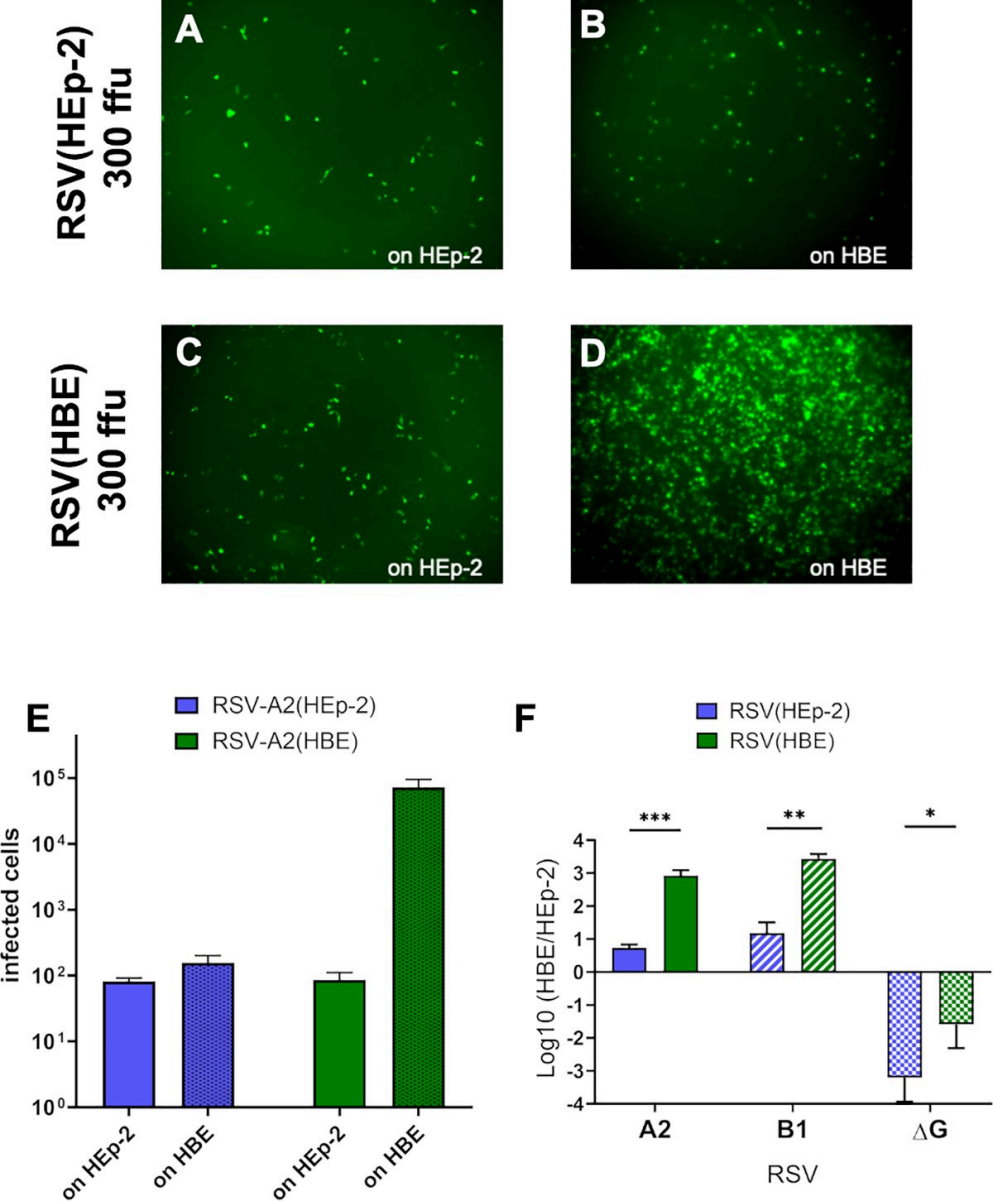
HBE grown RSV is significantly more infectious for HBE than for HEp-2 cells. HEp-2 and HBE were inoculated with 300 ffu (based on HEp-2 titration) of RSV-A2(HEp-2) and RSV-A2(HBE). Images of fluorescent cells after 24 h for RSV-A2(HEp-2) on HEp-2 (A) and on HBE (B). Images of fluorescent cells after 24 h for RSV-A2(HBE) on HEp-2 (C) and on HBE (D). Quantified infected cells of each virus inoculated with 100 ffu (based on HEp-2 titration) on HEp-2 and HBE (E). Relative infectivity was calculated by dividing the titer on HBE by that on HEp-2: HBE/HEp-2, mean values in Table 1 (F). Each virus stock was titrated on both HEp-2 and HBE on the same day, inoculating for 2 h at 37°C and counting fluorescent cells at 20 or 24 hpi, respectively. The mean and standard deviation (SD) of three independent experiments are plotted for F. (* p < 0.05, *** p < 0.001, **** p < 0.0001).

We investigated the infectivity of RSV produced in HBE [RSV(HBE)] to determine if virions containing LgG imparted an advantage for infection of HEp-2 or HBE. Both RSV(HEp-2) and RSV(HBE) viruses were titrated on HEp-2 cells and 300 ffu of RSV(HEp-2) or RSV(HBE) were used to inoculate HEp-2 and HBE. While RSV(HEp-2) infected both cell cultures similarly (Fig 3A and 3B), there were significantly increased number of infected cells in the HBE culture inoculated with RSV(HBE) (Fig 3C and 3D). We quantified the difference by titrating both viruses on HEp-2 and on HBE (Fig 3E) and using those values to determine their relative infectivity by dividing the viral titers on HBE by those on HEp-2 cells (HBE/HEp-2) (Fig 3F). The HBE/HEp-2 ratio was 876 for RSV-A2(HBE) and 6 for RSV-A2(HEp-2) (Table 1). We drew two conclusions from these results: 1) HBE cultures are more susceptible to RSV infection than HEp-2 with HBE/HEp-2 ratios > 1 for both viruses; and 2) RSV(HBE) is significantly more infectious for HBE than for HEp-2. Since HBE/HEp-2 is a relative measure of infectivity we cannot directly conclude that RSV(HBE), with LgG, has a major advantage in infecting HBE cultures. The alternative possibility is that RSV(HBE) infects HEp-2 cells poorly.

**Table 1:** Average HBE/HEp-2 values plotted in Fig 3F. Fold difference was calculated by dividing HBE/HEp-2 value for HBE grown virus, by that of HEp-2 grown virus.

	HBE/HEp-2		
	A2	B1	ΔG
**HEp-2 grown**	5.37	18.3	0.00180
**HBE grown**	876	2810	0.0542
*Fold-difference*	*163*	*154*	*30*

For both laboratory strains, RSV-A2 and RSV-B1, HBE grown virus has an HBE/HEp-2 ratio over 100-fold higher than HEp-2 grown virus (Fig 3F and Table 1). To determine if this higher infectivity on HBE was due to the large G (LgG) protein we performed the same experiment with RSV-ΔG (A2 virus lacking the G gene) which had been produced in HEp-2 or HBE. RSV-ΔG had a relative infectivity (HBE/HEp-2) significantly less than 1 (Fig 3F and Table 1). These results demonstrate the importance of G for efficient infection of HBE, consistent with in vivo studies where replication of virus lacking G cannot be detected [4,5]. Since virus lacking G is poorly infectious for HBE, G is likely responsible for differences in infectivity for RSV(HEp-2) and RSV(HBE).

### RSV(HBE) is less infectious for HEp-2 cells when assessed on a per genome basis

The G protein is responsible for RSV attachment to both HEp-2 and HBE, but the receptors that the G protein uses to bind on the two cell culture systems are different. The G glycoprotein utilizes heparan sulfate proteoglycans (HSPGs) on the surface of immortalized cells and likely utilizes CX3CR1 on HBE to initiate infection. The interacting site on the G protein with these two different receptors is also different (Fig 1C), and that difference might be relevant to the observed differences in infectivity.

**Fig 4:**
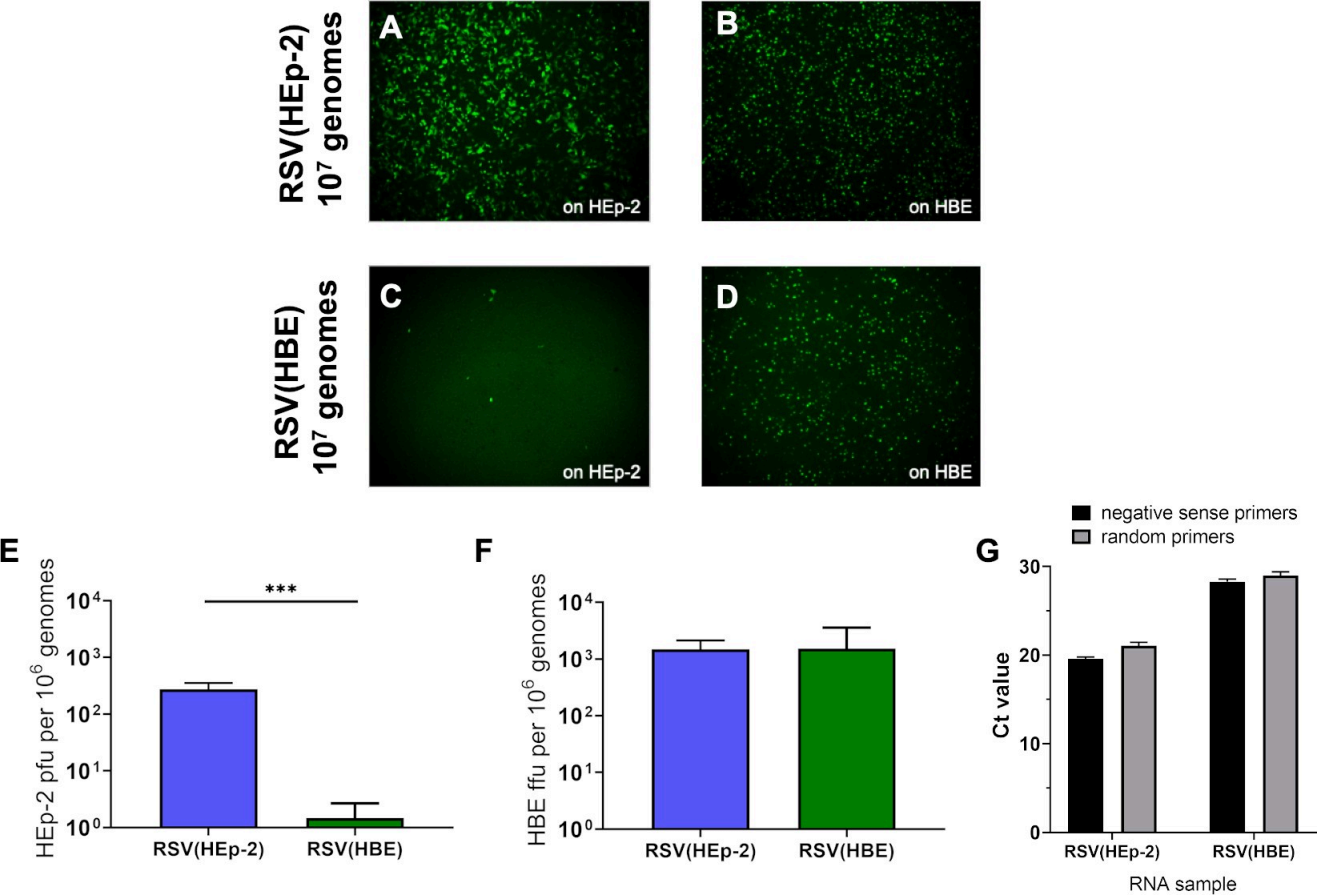
HBE-grown RSV virions are less infectious on HEp-2 cells when the comparison is based on genome numbers in the inoculum. Images of HEp-2 and HBE 24 h after inoculation of 10^7^ genomes of RSV(HEp-2) (A and B) and RSV(HBE) (C and D). Infectivity was determined by ffu per 10^6^ genomes on HEp-2 (E) and HBE (D). Viruses were titrated on both cultures to quantify ffu and RNA was extracted from the unused inoculum and genomes quantified by RT-qPCR for the RSV N (nucleocapsid) gene. The mean of three independent experiments are plotted. (*** p < 0.001). Comparison of Ct value when using negative sense specific versus random primers for reverse transcription (G).

The approach we used to assess the relative infectivity so far is missing an important component: the number of virions involved in each case. The number of virions present in each virus preparation can most accurately be determined by quantifying the genome copy numbers for each of virus preparation. To this end, we extracted the RNA genome from the same virus stocks that were used in the experiment shown in Fig 4E and 4F and used RT-qPCR targeting the nucleocapsid (N) protein gene to quantify the number of genomes. qPCR titer alone contains quantification of non-infectious and infectious virions but combined with infectious titer (ffu/mL) it provides information about how many genomes are present for each infectious virus. For simplicity, we expressed the result as “ffu per million genomes” for this comparison, allowing us to quantify the absolute infectivity of virions on HEp-2 and HBE separately. The RSV(HBE) virus had an average of 2 HEp-2 ffu per million genomes compared to 278 ffu per million genomes for RSV(HEp-2), clearly demonstrating that RSV(HBE) is poorly infectious for HEp-2 cells (Fig 4E). We confirmed that extracted RNA was genome specific and not contaminated by mRNA that may have been released from cell by comparing reverse transcription with negative sense specific versus random primers. Both methods resulted in similar Ct values Fig 4G). The result that RSV(HBE) is poorly infectious for HEp-2 cells is easily visualized by the number of infected cells observed when inoculated with virions containing 10^7^ genomes (Fig 4A–4C). The same amount of inoculum (10^7^ genomes) of both viruses was added to both HEp-2 and HBE. In 3 of the 4 situations efficient infection occurred, indicating that virus is not inhibited. In the fourth case, RSV(HBE) on HEp-2 cells, infection was much lower. If inhibition had been due to aggregation or another effect, it would have been consistent for RSV(HBE) on HEp-2 and HBE cultures since the same inoculum was used. Instead, using inoculum from the same virus and dilution tube, we observed high infection on HBE cultures and very low infection on HEp-2 cells. Since RSV(HBE) is much less infectious for HEp-2 cells, its titer on HEp-2 cells is not an accurate reflection of its infectivity in HBE and therefore in vivo. This result explains why we had observed many more infected HBE cells than HEp-2 cells when inoculating with RSV(HBE) and RSV(HEp-2) whose infectivity had been determined on HEp-2 cells (Figs 3C, 3D, 4C and 4D).

RSV with mutations in the CX3C motif of the G glycoprotein have reduce infectivity for HBE, indicating the mechanism for viral attachment in HBE includes this domain [7–10] (Fig 1C). When assayed on HBE, RSV has a similar number of ffu per million genomes independent of the cell type the virus was produced in, approximately 1 ffu/10^3^ genomes (Fig 4F). This result indicates that the HBE receptor binding ability of the G protein in RSV(HBE) and RSV(HEp-2) virion preparations is equivalent in initiation of HBE infection. Therefore, LgG found in RSV(HBE) functions equivalently to the G found in RSV(HEp-2) in terms of receptor binding, leading to initiation of HBE infection (Fig 4B–4D). However, the increase in G protein size, from G to LgG had a profoundly negative effect on LgG protein function in HEp-2 cell infection, most likely interfering with the ability of its HBD motif to bind to HSPG on the surface of HEp-2 cells.

### HBE grown virus is less dependent on HSPG for infecting HEp-2

The finding that the RSV(HBE) with LgG is less infectious for HEp-2 cells suggests that the structural modification leading to the increased size of LgG inhibits the attachment mechanism for HEp-2 cells. The virion G protein binds to HSPG on the surface of HEp-2 and other immortalized cells to initiate infection [24–27] (Fig 1C). We hypothesized that RSV(HBE) does not use HSPG which are decorated with glycosaminoglycans (GAGs) as efficiently as RSV(HEp-2) which could explain their lower infectivity in cells in which infectivity is largely dependent on HSPGs. To test the ability of viruses to efficiently use GAGs, we compared their infectivity for cells expressing GAGs compared to cells deficient in GAGs. CHO-K1 express GAGs and CHO-A745 cells are GAG deficient. The CHO-A745 cells are deficient in xylosyl transferase which adds the first sugar, xylosyl, to a particular cell surface proteins to initiate a long HS polymer addition [23].

**Fig 5:**
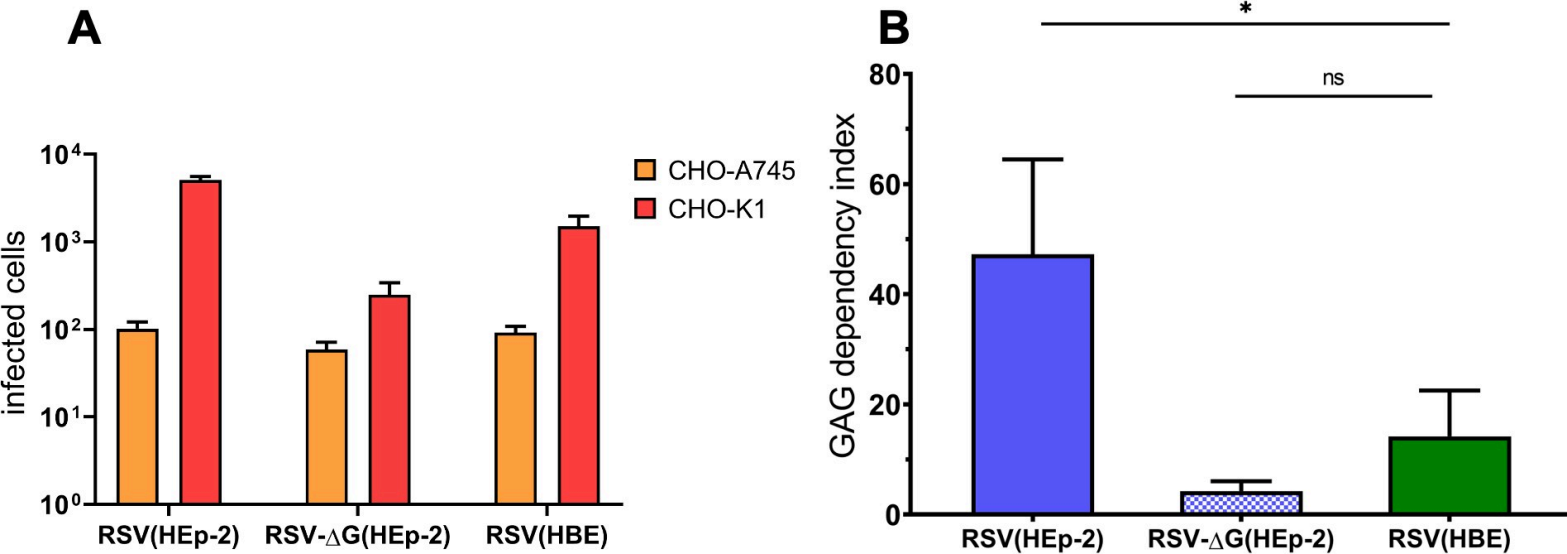
GAG dependency of RSV(HEp-2), RSV(HBE) and RSV-ΔG(HEp-2). RSV A2 and ΔG viruses grown on HEp-2 or HBE) were titrated on CHO-K1 and CHO-A745. A representative titration experiment (A). The number of foci on CHO-K1 was divided by that on CHO-A745 to calculate the GAG dependency index from three independent experiments (B). (* p < 0.05).

The “GAG dependency index” quantifies the contribution of the GAGs, linked to HSPG, to the initiation of virus infection by comparing virus infectivity on CHO-K1 cells divided by the infectivity of the same virus on CHO-A745 cells [19]. The average GAG dependency index of RSV(HBE) was determined to be 17 and RSV(HEp-2) to be 47 (Fig 5). RSV(HBE) has some dependence on GAGs but this dependence is significantly lower than that of RSV(HEp-2).

RSV-ΔG(HEp-2) also displays a GAG dependency of 4.2, also low. Some GAG dependency is expected for RSV-ΔG due to the ability of the fusion protein, F, to interact with HSPG on the cell surface [28]. However, most of the contribution of RSV and HSPG interaction is through the G glycoprotein. The decreased dependency that RSV(HBE) has on the HSPG GAGs provides a plausible explanation for why RSV(HBE) is poorly infectious for HEp-2 cells: the modification that results in the larger size of LgG prevents it from binding HSPG, presumably by occluding the heparan binding domain (HBD) of G in some way.

### Clinical RSV samples passaged on HBE cultures have LgG and are less infectious for HEp-2 cells

**Fig 6:**
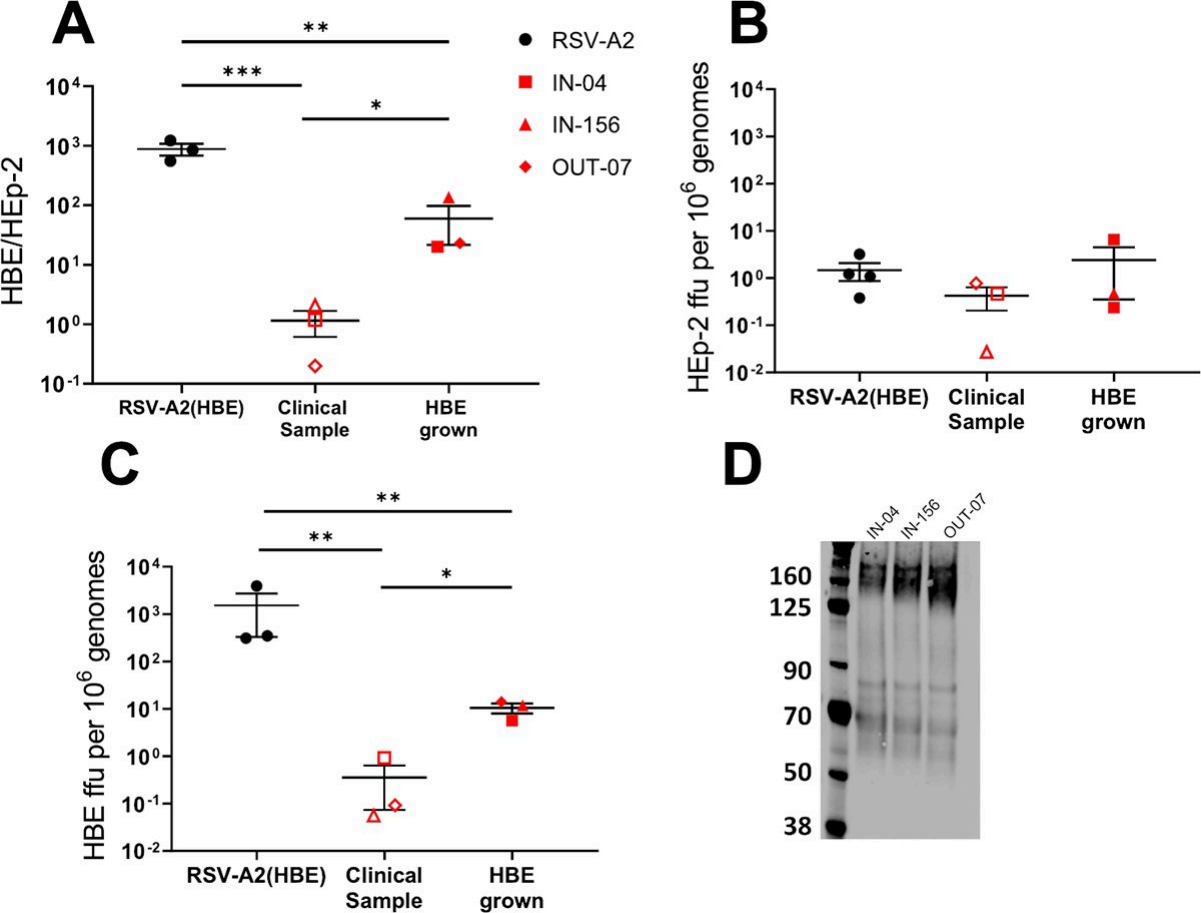
Infectivity of original and HBE grown clinical RSV samples on HEp-2 and HBE cultures. Clinical samples were passaged on HBE cultures once to produce HBE grown viruses. A) HBE/HEp-2 ratio based on titration of nasal wash samples or HBE grown virus on HBE and HEp-2 cells. B) HEp-2 ffu per 10^6^ genomes and C) HBE ffu per 10^6^ genomes, based on infectious virus titration and RT-qPCR analysis from the same inoculum. D) Immunoblot of G of the virus produced from HBE cultures infected with each of the clinical nasal wash or nasopharyngeal samples.

Since HBE cultures are an excellent ex vivo model of the human bronchial epithelium that RSV infects, we hypothesized that the RSV that is produced in vivo in the respiratory samples of infected individuals would behave similarly to RSV(HBE). We sought to characterize the infectivity of clinical RSV samples and compare them to what we find for RSV(HBE): higher infectivity on HBE cultures than HEp-2 cells. Although the clinical RT-PCR titer was considered high for these samples (10^8^ genomes/mL), for the infectivity experiments this concentration and volume was too low to perform repeat infectivity experiments from the same samples and so we drew conclusions from three different clinical samples instead. Frozen clinical samples were thawed then diluted and incubated at 37°C for 1 hour prior to inoculation in an attempt to hydrate the mucus and allow the RSV to separate from the mucus and debris present in sample. Surprisingly, low levels of RSV infectivity were detected in these clinical samples and their infectivity was not dramatically higher for HBE than HEp-2, HBE/HEp-2 of >100, as we had observed for the A2 lab strain RSV(HBE) (Fig 6). These clinical samples were all less than 2-fold higher. In fact, sample OUT-07 was more infectious on HEp-2 than on HBE.

To determine and compare infectivity on each cell type directly, ffu per 10^6^ genomes were calculated for HEp-2 and HBE (Fig 6B and 6C). Here, we find low infectivity for both HEp-2 and HBE cultures, different from RSV(HBE) which had low infectivity only on HEp-2 cells (Fig 6B). These results are surprising, given how well the HBE model reflects the in vivo environment and will be explored in the Discussion.

To further examine the unexpectedly similar infectivity of the clinical nasal wash on HBE and HEp-2 cultures, we passaged each sample in HBE cultures by infecting and collecting apical wash and repeated the infectivity comparison. After one passage on HBE cultures, the G protein of all three clinical isolates was in the LgG form of 170 kDa (Fig 6D), similar to the laboratory A2 strain and the virus produced in HBE was more infectious for HBE than HEp-2 (Fig 6) including when calculated as ffu per million genomes on HBE (Fig 6C). However, the HBE/HEp-2 ratio is approximately 10-fold lower for the clinical samples compared to the laboratory A2 strain. Although we detect infection of these clinical samples by immunofluorescent staining instead of GFP expression, infection is observed at similar timepoint meaning both techniques are equally sensitive. Based on the ffu per million genomes results, the HBE grown clinical viruses are more infectious for HBE compared to the original clinical sample, with no significant difference in their ability to infect HEp-2 cells.

Although the magnitude of difference between HBE infectivity is less for these HBE grown clinical RSV samples, the characteristic of higher infectivity on HBE and lower infectivity for HEp-2 cells is conserved along with presence of LgG. These clinical viruses were only passaged one time in HBE cultures to ensure that they would be as genetically similar to the original sample as possible. Altogether, these results demonstrate that virus grown on HBE cultures, whether from a lab-adapted strain or original clinical samples, results in a virus that contains LgG and is less infectious for HEp-2 cells.

## Discussion

HBE cultures are an ex vivo model for RSV infection and pathogenesis that mimics the in vivo target of RSV, the human airway epithelium, more closely than the immortalized tumor cell lines traditionally used to study RSV infection. We previously found that RSV virions produced by HBE cultures, RSV(HBE), carry a 170 kDa form of the G glycoprotein, LgG, rather than the 95 kDa G protein in RSV(HEp-2) virions [19]. Here we demonstrate that this size differential holds for both RSV subgroups, A and B, and for lab-adapted strains and clinical isolates of RSV. While RSV(HEp-2) infects HEp-2 and HBE cultures with similar efficiency, RSV(HBE) with its LgG is 100-fold more infectious for HBE cultures than for HEp-2. Surprisingly, genome quantification revealed that the 100-fold higher infectivity of RSV(HBE) for HBE cultures than for HEp-2 cells is due to its 100-fold lower infectivity for HEp-2 cells. The modification of the G protein responsible for the larger size of LgG is not known, but the presence of this modification of LgG in virions does not inhibit their ability to infect HBE cultures. The modification that produces LgG, therefore, appears to interfere with the function of the heparin binding domain (HBD) of the G protein, likely preventing its attachment to the HSPGs of HEp-2 cells (Fig 1). Consistent with this possibility, RSV(HBE) displayed reduced infectivity for CHO cells relative to its infectivity for HSPG-deficient CHO cells, indicating decreased usage of HSPGs as a receptor. These results are consistent with the finding that HSPGs are not present on the apical surface of HBE cultures [6] and therefore could not function as the receptor for RSV on the ciliated cells of HBE cultures.

The RSV G protein, like the G protein of the related human metapneumovirus, is not essential for infection of immortalized cell lines such as HEp-2. RSV(HEp-2) virions lacking G protein retain 30% the infectivity of RSV containing the G protein [29,30]. However, here we found that on HBE cultures, RSV(HEp-2) virions lacking G protein retain only 0.1% the infectivity of virions containing the G protein (Fig 3F). The poor infectivity of RSV lacking G (RSV-ΔG) for HBE cultures is reminiscent of in vivo studies that did not detect viral replication in mice inoculated with RSV-ΔG [4,5]. The large (~1,000-fold) reduction of RSV-ΔG infectivity for HBE cultures highlights how much more critical G is for infection of HBE cultures, and therefore likely in vivo, than in immortalized cells, the cells that have been most frequently used to study RSV.

The dramatically higher contribution that the G protein makes to RSV(HEp-2) infectivity for HBE cultures (~1,000-fold) than for HEp-2 cells (3-fold) is likely related to the different receptors it uses to attach to these two cell types. The RSV G protein receptor on HBE ciliated cells is likely CX3CR1 [7–9] which would bind to the CX3C motif in the G protein, whereas the G protein receptor on HEp-2 cells is HSPG where the interaction is relatively non-specific, between the positively charged residues in the HBD of the G protein and the negatively charged sulfate groups of the HSPG heparan sulfate chains. Such major differences in the initiation of infection could affect assays designed to quantify neutralizing antibodies against the G protein. For instance, antibodies against G that neutralize RSV infectivity for HEp-2 and other immortalized cell lines would likely bind to the HBD of the G protein, while antibodies against G that neutralize RSV in HBE cultures and in vivo would likely bind to the CX3C region of G. Conversely, many antibodies that block the CX3C region would not affect the HBD, thereby inhibiting infection of HBE cultures but not infection of HEp-2 cells. This possibility has been demonstrated by the well characterized monoclonal antibody, 131-2G, against the CX3C motif region the that blocks infection in vivo and in HBE cultures, but not in immortalized cells [7,31]. HBE cultures, therefore, may provide a more accurate definition of antibodies to G that would neutralize in vivo.

LgG is approximately twice the size of the G protein produced in immortalized cells which suggests at least two hypotheses for the size differential between G and LgG. One hypothesis is that LgG is a covalent dimer of G. A disulfide dimer is unlikely, since LgG retains its large size under reduced conditions [19]. There are at least two intermolecular cross-linking enzymes that covalently link homodimers in eukaryotic cells: transglutaminases and tyrosinases [32]. Transglutaminases are present in many different cell types, but are not present in the ER or Golgi where G is processed [33].

A second hypothesis is that increased O-linked glycosylation of G results in LgG. O-linked glycan addition to Ser or Thr is the major post-translational modification of the G protein produced in HEp-2 cells, adding an estimated 35 or more glycans to the protein [34]. There are a total of 69 Ser and Thr residues in the ectodomain (aa67-298) of the A2 G protein. Some of the 34 Ser and Thr residues that are not modified in HEp-2 cells could be modified by O-linked glycans in HBE cultures. It is also possible that additions are made to the existing O-linked glycan structures making them larger and more complex when produced in HBE cultures. It is also possible that GAGs are linked to some of these Ser or Thr residues when they are produced in HBE cultures. Finally, a combination of these possibilities could be responsible for the increased size.

As described above, the difference in size between G and LgG proteins could be caused by either dimerization of the G protein or by more extensive O-linked glycosylation. Whichever it is, these same modifications impact the HBD interaction with HSGP required for infection of immortalized cells. They might also sterically hinder B-cell education/binding and therefore the production of antibodies to the G protein. Steric hinderance by these additional glycans could help to explain why antibodies against G are present in serum at much lower concentrations than antibodies to the F protein [35]. Currently, the most prevalently circulating strains of RSV, BA and ON-1, both express G proteins with duplications in the C-terminal hypervariable region of G of 24 and 20 amino acids, respectively [36,37]. Each of these duplicated regions add 9 or more potential O-glycosylation sites perhaps further shielding the G protein from antibodies and providing greater resistance to neutralization of RSV virions by antibodies.

In this report we have characterized the appearance of a much larger, LgG protein, in RSV(HBE) virions, produced by primary HBE cultures. However, the in vivo biological relevance of LgG in naturally circulating RSV remains unclear. The infectivity of the virus from nasal wash and nasopharyngeal swab samples contained at least 10-fold lower amount of virus by RT-qPCR (10^8^–10^9^ genomes/mL) compared to virus collected from that collected from HBE cultures. The virus in these clinical samples displayed low specific infectivity (0.4 ffu per million genomes). They did not display the reduced HEp-2 infectivity (relative to HBE cultures) phenotype that we had found with RSV(HBE): low infectivity on HEp-2 cells (~2 ffu per million genomes) and high infectivity on HBE cultures (~1000 ffu per million genomes). Instead, they showed low infectivity for both cell types. However, one passage in HBE cultures resulted in virions with LgG and the low HEp-2 cell infectivity and increased infectivity on HBE cultures.

Nasal wash or swab samples are routinely collected from patients to identify a viral pathogen by RT-PCR. These sampling procedures are adequate for clinical needs because functional virions are not required for RT-PCR and only a few functional virions are needed to initiate growth of the virus in culture. However, these procedures are not optimized to examine infectivity of the samples as performed in these experiments. For example, virus samples are not snap-frozen on dry ice which is routinely used to preserve virus infectivity in our laboratory. Additionally, the RSV collected in the clinical samples may be altered by their exposure to the inflammatory environment of the immune response in the respiratory tract of infected infants. Although HBE cultures mimic the in vivo epithelium, they lack the cellular immune response to RSV infection in the respiratory tract. This cellular immune response likely exposes the virions to proteases released from dead epithelial and immune cells and microbial byproducts, all of which could degrade virion proteins thereby reducing infectivity. Considering these limitations that likely contribute to the low infectious yield of the clinical samples, it is difficult to determine if RSV in nasal secretions is initially similar to RSV(HBE) before being exposed to the inflammatory environment.

In this report we have partially characterized the function of a much larger form of the RSV G attachment glycoprotein, LgG, produced by primary HBE cultures. We found that LgG in virions efficiently mediates infection of HBE cultures, but these virions have lost much of their ability to infect HEp-2 cells. These results highlight the role of the cell, particularly the natural target cell, expressing a viral protein in a manner that affects its function, infectivity, in a different target cell where it uses a different virus receptor. These differences may affect the interpretation of virus infectivity, the mechanisms of entry, the ability to isolate virus from patient samples and possibly the quantification of neutralizing antibodies. While the HBE cultures used here contain the natural in vivo target cells for RSV, and the immortal HEp-2 tumor cell line does not, it seems possible that a virus infecting one cell type in the body might experience modifications that affect their ability to infect a second cell type.
